# Efficacy and safety of endothelin A receptor antagonists in IgA nephropathy: a systematic review and meta-analysis

**DOI:** 10.1093/ckj/sfaf066

**Published:** 2025-02-27

**Authors:** Zhonghua Tian, Yalin Yang, Jixiong Mei, Mingchun Huang, Yanyan Li, Zhie Fang, Yunyi Li, Ling Tang, Yuxia Li

**Affiliations:** Department of Pharmacy, Chongqing Hospital of Traditional Chinese Medicine, Chongqing, China; Department of Pharmacy, Chongqing Hospital of Traditional Chinese Medicine, Chongqing, China; Department of Pharmacy, Chongqing Hospital of Traditional Chinese Medicine, Chongqing, China; Department of Pharmacy, Chongqing Hospital of Traditional Chinese Medicine, Chongqing, China; Department of Pharmacy, Chongqing Hospital of Traditional Chinese Medicine, Chongqing, China; Department of Pharmacy, Chongqing Hospital of Traditional Chinese Medicine, Chongqing, China; Department of Pharmacy, Chongqing Hospital of Traditional Chinese Medicine, Chongqing, China; Department of Pharmacy, Chongqing Hospital of Traditional Chinese Medicine, Chongqing, China; Department of Nuclear Medicine, Chongqing Emergency Medical Center, Chongqing, China

**Keywords:** atrasentan, endothelin A receptor antagonist, IgA nephropathy, SC0062, sparsentan

## Abstract

**Background:**

Immunoglobulin A nephropathy (IgAN) is the most common primary glomerulonephritis worldwide. Endothelin A receptor activation is a key driver of proteinuria, inflammation and fibrosis in IgAN. This systematic review and meta-analysis aimed to comprehensively evaluate the efficacy and safety of endothelin A receptor antagonists (EARAs) in IgAN patients.

**Methods:**

PubMed, Embase, Web of Science and Cochrane Library were searched from inception to 31 October 2024. All randomized controlled trials were identified according to the inclusion criteria. Data were analyzed by RevMan 5.4.

**Results:**

Four high-quality studies were included, comprising 1346 IgAN patients. Compared with the control group, EARAs group achieved a greater reduction in urine protein–creatinine ratio (UPCR) [mean difference (MD) –31.89, 95% confidence interval (CI) –37.50 to –26.28], systolic blood pressure (BP) (MD –2.78, 95% CI –4.11 to –1.44) and diastolic BP (MD –4.12, 95% CI –5.24 to –2.99), and a smaller reduction in estimated glomerular filtration rate (eGFR) (MD 4.10, 95% CI –0.76 to 8.96). However, the EARAs group had higher risk of anemia [odds ratio (OR) 2.38, 95% CI 1.54 to 3.69], cough (OR 2.27, 95% CI 1.24 to 4.15), dizziness (OR 2.37, 95% CI 1.51 to 3.71), hypotension (OR 2.39, 95% CI 1.56 to 3.67), fluid retention (OR 1.46, 95% CI 1.04 to 2.05) and acute kidney injury (OR 3.12, 95% CI 1.31 to 7.42).

**Conclusion:**

EARAs can significantly reduce UPCR, lower both systolic and diastolic BP, and delay eGFR decline in IgAN patients. However, they may cause anemia, cough, dizziness, hypotension, fluid retention and acute kidney injury.

## Graphical Abstract

**Graphical Abstract:**
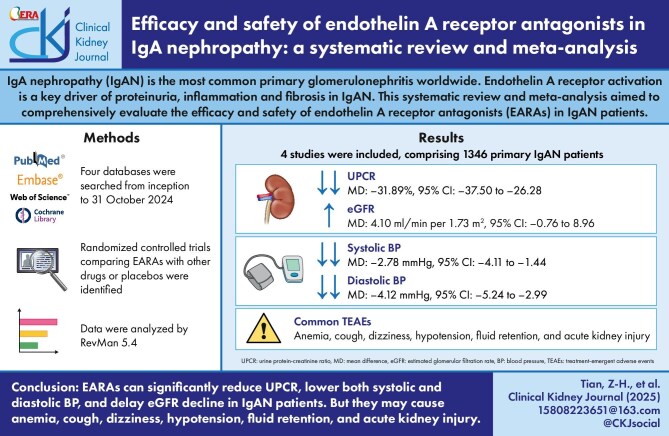

KEY LEARNING POINTS **What was known:**

- Immunoglobulin A nephropathy (IgAN) is the most common primary glomerulonephritis worldwide.
- Endothelin A receptor activation is a key driver of proteinuria, inflammation and fibrosis in IgAN.

**This study adds:**

- Endothelin A receptor antagonists (EARAs) can significantly reduce urine protein–creatinine ratio, lower both systolic and diastolic blood pressure, and delay the decline of estimated glomerular filtration rate in IgAN patients.
- EARAs may be associated with anemia, cough, dizziness, hypotension, fluid retention and acute kidney injury.

**Potential impact:**

- This study supports the inclusion of EARAs as a treatment option for IgAN patients.
- This study sets the stage for larger, high-quality randomized controlled trials to explore the long-term efficacy and safety of EARAs in IgAN patients.

## INTRODUCTION

Immunoglobulin A nephropathy (IgAN) is the most common primary glomerulonephritis worldwide, with an incidence rate of at least 2.5/100 000 adults per year [1–4]. Due to the progressive nature of disease, IgAN patients often face a poor prognosis if the condition is not properly controlled [3]. Research has revealed that although IgAN can be a slowly progressive disease, in the absence of effective treatment, approximately 30%–40% of patients will progress to end-stage renal disease (ESRD) within 10 years, increasing to >50% within 20 years [3, 5–7]. Upon progression to ESRD, patients will encounter a profound decrement in quality of life, a substantial escalation in healthcare costs and a significant increase in risk of mortality. Therefore, effective treatments to delay the progression of IgAN are urgently needed.

Studies find that the main risk factors for disease progression in IgAN is decreased glomerular filtration rate (GFR), persistent proteinuria and hypertension, each of which is an independent risk factor [4, 8, 9]. Therefore, the current treatment of IgAN mainly focus on supportive treatment, including smoking cessation, alcohol restriction, weight management and other lifestyle interventions, along with the use of renin–angiotensin system inhibitors, aiming to reduce proteinuria, lower blood pressure (BP), and delay the decline of GFR. However, despite optimized supportive treatment, many IgAN patients remain at high risk of rapid progression, a significant characteristic of which is urinary protein excretion >1 g/day. For patients who remain at high risk of rapid progression and whose estimated GFR (eGFR) is >50 mL/min/1.73 m^2^ after 3–6 months of optimized supportive therapy, the Kidney Disease: Improving Global Outcomes (KDIGO) guidelines suggest the use of systemic corticosteroids, and for Chinese patients, mycophenolate mofetil can be added to reduce the dosage of corticosteroids, but their safety profiles should not be ignored [2, 10, 11]. Other immunosuppressants commonly used in primary or secondary glomerulonephritis, such as cyclophosphamide, calcineurin inhibitors and rituximab, are not recommended for the treatment of IgAN due to their poor efficacy [2]. Therefore, for a long time, the treatment options for IgAN have been very limited, and exploring new safe and effective treatment methods to improve the therapeutic dilemma of IgAN remains a research hotspot.

Endothelin-1, a vasoactive peptide involved in the pathophysiology of IgAN, has been found to play a key role in endothelial dysfunction, podocyte dysfunction, mesangial expansion, tubular injury, inflammation and fibrosis through activation of endothelin A receptors in experimental models of IgAN [12–15]. In recent years, a series of randomized controlled trials (RCTs) have successively confirmed that endothelin A receptor antagonists (EARAs), such as atrasentan, sparsentan and SC0062, could effectively treat IgAN, and no fatal cases were reported [13, 16, 17]. Among them, sparsentan has been approved by the Food and Drug Administration to treat IgAN [18]. However, until now, there have been no studies comprehensively analyzing the efficacy and safety of EARAs in IgAN patients. Therefore, we adopted the meta-analysis method for the first time to comprehensively evaluate the efficacy and safety of EARAs in IgAN patients, aiming to provide evidence for the clinical use of these drugs.

## MATERIALS AND METHODS

This study was conducted according to the Preferred Reporting Items for Systematic Reviews and Meta-analyses (PRISMA) statement, and the study protocol was registered in PROSEPRO database (CRD42024615381).

### Search strategy

PubMed, Embase, Web of Science and Cochrane Library were systematically searched from inception to 31 October 2024. The following keywords were applied: (‘endothelin A receptor antagonist’ OR ‘endothelin receptor antagonist’ OR ‘endothelin’ OR ‘atrasentan’ OR ‘sparsentan’ OR ‘SC0062’ OR ‘ambrisentan’ OR ‘sitaxsentan’ OR ‘bosentan’ OR ‘tezosentan’ OR ‘zibotentan’ OR ‘clazosentan’ OR ‘aprocitentan’ OR ‘macitentan’ OR ‘avosentan’ OR ‘darusentan’) AND (‘Immunoglobulin A nephropathy’ OR ‘IgA nephropathy’ OR ‘IgAN’).

### Study selection

Studies were eligible for inclusion if the following criteria were met: (i) the study was published in English language; (ii) the study was published in a peer-reviewed journal; (iii) the study population was adult patients (≥18 years old) with biopsy-proven IgAN; (iv) the research type was an RCT; (v) the intervention strategy compared EARAs with other drugs or placebos; (vi) the study lasted at least 24 weeks; (vii) the study reported at least one required renal outcome or treatment-emergent adverse events (TEAEs).

All conference papers, reviews, case reports, case series reports, animal experiments, *in vitro* studies, duplicate studies, non-RCT studies, studies where key data could not be extracted and non-English studies were excluded.

### Outcomes

The primary outcomes included the change from baseline in urine protein–creatinine ratio (UPCR), eGFR and blood pressure after treatment. The secondary outcomes included the proportion of patients achieving UPCR reduction ≥30%, ≥40% or ≥50% from baseline, achieving complete (<0.3 g/day) or partial (<1.0 g/day) proteinuria remission, and reaching the composite kidney failure endpoint [confirmed 40% eGFR reduction, end-stage kidney disease (defined as sustained eGFR <15 mL/min/1.73 m^2^, or initiation of renal replacement therapy) or all-cause mortality] after treatment. Any TEAEs and the influences of EARAs on hemoglobin, serum potassium, body weight, and B-Type Natriuretic Peptide (BNP) were also analyzed.

### Data extraction and bias risk assessment

Literature search, review and data extraction were conducted independently by two investigators (Y.Y. and J.M.) according to the study protocol. Any discrepancies were solved by discussion with each other or a third investigator (M.H.) if required. Data were extracted in detail, including study characteristics (first-author name, publication year and clinical trial number), population characteristics (sample size, gender, mean age and duration of disease), clinical characteristics of participants in baseline (body weight, BP, BNP, hemoglobin, serum potassium, eGFR and UPCR), research type, intervention methods (drug name, dosage, route of administration, frequency of administration and duration of treatment), duration of follow-up, relevant outcomes after treatment,and all TEAEs.

The risk of bias was independently assessed by two investigators (Yan.Li and Z.F.) using the Cochrane Risk of Bias 2 tool [19] in six domains, including bias in random sequence generation, allocation concealment, blinding of participants and researchers, blinding of outcome assessment, incomplete outcome data, and selective reporting. Any disagreements were solved by discussion with each other or a third investigator (Yun.Li) if needed.

### Statistical analysis

Statistical analysis was performed using RevMan 5.4. Changes from baseline in UPCR, eGFR, BP, BNP, hemoglobin, serum potassium and body weight after treatment were presented as mean difference (MD) with 95% confidence interval (CI). The proportion of patients achieving UPCR reduction, achieving complete or partial proteinuria remission, reaching composite kidney failure endpoint and experiencing TEAEs after treatment was reported as odds ratio (OR) with 95% CI. The *I*^2^ statistic was used to assess the statistical heterogeneity among studies. The heterogeneity of *I*^2^ was quantified as low, moderate and high with upper limits of 25%, 50% and 75%, respectively. When the value of *I*^2^ was ≥50%, significant heterogeneity was considered and a random-effect model was used. Otherwise, a fixed-effect model was adopted. Publication bias was evaluated using Egger test and Begg-Mazumdar test. *P* < .05 was considered statistically significant.

## RESULTS

### Literature search and study characteristics

**Figure 1:**
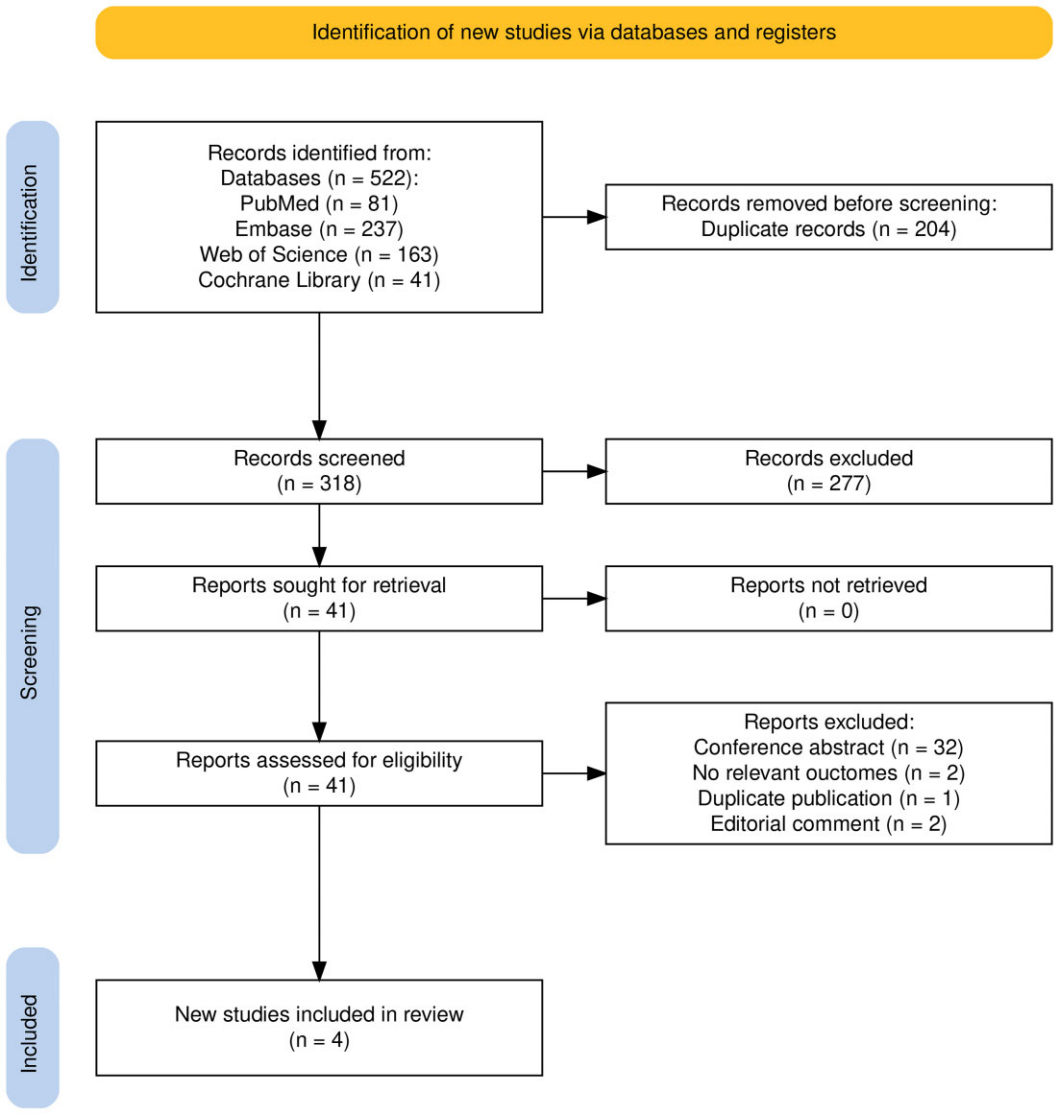
PRISMA flow diagram of the study selection process.

In the initial literature screening, a total of 522 studies were searched, of which 204 studies were removed due to duplication and 318 studies were further screen. After reading the title and/or abstract, 277 studies were removed for not meeting the inclusion criteria. Then, 41 studies were further assessed by reading the full text. Finally, four studies (including 1346 IgAN patients) met the inclusion criteria and were selected for the meta-analysis [13, 16, 17, 20]. Among the four studies, two studies were derived from the same clinical trial [16, 20]. However, the discrepancies in baseline characteristics of patients suggested that the populations included in the two studies were not completely consistent. Therefore, they were considered as two independent studies. Similarly, another study evaluated the therapeutic effects of three different doses of EARAs in IgAN patients [17]. Given that the aim of our study was to comprehensively evaluate the therapeutic effect of EARAs in IgAN patients, rather than the effect of drug doses differences on treatment outcomes, all three treatment regimens met our inclusion criteria. Therefore, for the convenience of statistical analysis, the study was also regarded as three independent studies. The process of literature search and selection is shown in Fig. 1.

For the patient characteristics of the included studies, three studies enrolled patients with proteinuria ≥1.0 g/day and an eGFR ≥30 mL/min/1.73 m^2^ [13, 16, 20], one study enrolled patients with UPCR ≥750 mg/g or proteinuria ≥1.0 g/day and an eGFR ≥30 mL/min/1.73 m^2^ [17]. In terms of drug intervention, one study compared atrasentan with placebo [13], two studies compared sparsentan with irbesartan [16, 20] and one study compared SC0062 with placebo [17]. The duration of follow-up ranged from 36 weeks to 110 weeks. All four studies reported the drug's safety and primary renal outcomes in whole or in part. The detailed characteristics of patients are presented in Table 1.

**Table 1:** Baseline characteristics of studies and participants included.

	Heerspink 2023 [16]	Rovin 2023 [20]	Heerspink 2024a [13]	Heerspink 2024b [17]	Heerspink 2024c [17]	Heerspink 2024d [17]
Study design	Randomized, double-blind, active-controlled study	Randomized, double-blind, active-controlled study	Randomized, double-blind, placebo-controlled study	Randomized, double-blind, placebo-controlled study	Randomized, double-blind, placebo-controlled study	Randomized, double-blind, placebo-controlled study
NCT number	NCT03762850	NCT03762850	NCT04573478	NCT05687890	NCT05687890	NCT05687890
Research institution	134 sites in 18 countries	134 sites in 18 countries	133 sites in 20 countries	46 centers in China	46 centers in China	46 centers in China
Population characteristics	Adults (aged ≥18 years) with biopsy-proven primary IgAN, a proteinuria of at least 1 g/day and an eGFR of at least 30 mL/min/1.73 m^2^	Adults (aged ≥18 years) with biopsy-proven primary IgAN, a proteinuria of at least 1 g/day and an eGFR of at least 30 mL/min/1.73 m^2^	Adults (aged ≥18 years) with biopsy-proven primary IgAN, a proteinuria of at least 1 g/day and an eGFR of at least 30 mL/min/1.73 m^2^	Adults (aged ≥18 years) with biopsy-proven primary IgAN, a proteinuria of at least 1 g/day or UPCR of at least 750 mg/g, and an eGFR of at least 30 mL/min/1.73 m^2^	Adults (aged ≥18 years) with biopsy-proven primary IgAN, a proteinuria of at least 1 g/day or UPCR of at least 750 mg/g, and an eGFR of at least 30 mL/min/1.73 m^2^	Adults (aged ≥18 years) with biopsy-proven primary IgAN, a proteinuria of at least 1 g/day or UPCR of at least 750 mg/g, and an eGFR of at least 30 mL/min/1.73 m^2^
Intervention measure	Intervention group: sparsentan, 400 mg once daily, control group: irbesarten, 300 mg once daily	Intervention group: sparsentan, target dose 400 mg once daily, control group: irbesartan, target dose 300 mg once daily	Intervention group: atrasentan, 0.75 mg once daily, control group: placebo	Intervention group: SC0062, 5 mg once daily, control group: placebo	Intervention group: SC0062, 10 mg once daily	Intervention group: SC0062, 20 mg once daily
Duration of follow-up (weeks)	36	110	36	24	24	24
Sample size (*n*)	Intervention group: 202, control group: 202	Intervention group: 202, control group: 202	Intervention group: 135, control group: 135	Intervention group: 33, control group: 34	Intervention group: 32, control group: 34	Intervention group: 32, control group: 34
Sex, female (*n*)	Intervention group: 63, control group: 59	Intervention group: 63, control group: 59	Intervention group: 54, control group: 57	Intervention group: 16, control group: 11	Intervention group: 21, control group: 11	Intervention group: 18, control group: 11
Age (years), mean ± SD	Intervention group: 46.6 ± 12.8, control group: 45.4 ± 12.1	Intervention group: 46.6 ± 12.8, control group: 45.4 ± 12.1	Intervention group: 45.7 ± 12.9, control group: 44.1 ± 11.0	Intervention group: 43 ± 11, control group: 43 ± 13	Intervention group: 40 ± 11, control group: 43 ± 13	Intervention group: 43 ± 11, control group: 43 ± 13
Duration of disease (years), mean ± SD	Intervention group: 6.4 ± 6.5, control group: 6.4 ± 7.1	Intervention group: 4.0 (1.0–10.0), control group: 4.0 (1.0–10.0)	Intervention group: 5.14 ± 5.41, control group: 6.14 ± 6.04	Intervention group: 3.5 (0.5–7.5), control group: 3.0 (1.2–8.0)	Intervention group: 3.6 (2.5–6.5), control group: 3.0 (1.2–8.0)	Intervention group: 3.0 (1.0–5.5), control group: 3.0 (1.2–8.0)
BMI (kg/m^2^), mean ± SD	-	-	Intervention group: 27.05 ± 5.44, control group: 27.66 ± 4.46	Intervention group: 24.5 ± 3.0, control group: 24.8 ± 4.1	Intervention group: 24.3 ± 5.7, control group: 24.8 ± 4.1	Intervention group: 24.6 ± 3.4, control group: 24.8 ± 4.1
SBP (mmHg), mean ± SD	Intervention group: 128.0 ± 14.4, control group: 130.0 ± 12.4	Intervention group: 128.0 ± 14.4, control group: 129.9 ± 12.4	Intervention group: 125.4 ± 13.3, control group: 122.9 ± 12.3	Intervention group: 119 ± 11, control group: 119 ± 15	Intervention group: 119 ± 13, control group: 119 ± 15	Intervention group: 120 ± 15, control group: 119 ± 15
DBP (mmHg), mean ± SD	Intervention group: 82.0 ± 10.6, control group: 83.0 ± 10.6	Intervention group: 81.6 ± 10.6, control group: 83.2 ± 10.6	Intervention group: 79.6 ± 9.8, control group: 78.7 ± 9.0	Intervention group: 75 ± 8, control group: 76 ± 10	Intervention group: 77 ± 8, control group: 76 ± 10	Intervention group: 77 ± 10, control group: 76 ± 10
Urinary protein excretion (g/day)	Intervention group: 1.8 (1.2–2.8), control group: 1.8 (1.3–2.6)	Intervention group: 1.8 (1.2–2.9), control group: 1.8 (1.3–2.6)	Intervention group: 1.8 (1.3–2.8), control group: 1.9 (1.3–2.6)	-	-	-
24-h UPCR (g/g)	Intervention group: 1.3 (0.8–1.8), control group: 1.2 (0.9–1.7)	Intervention group: 1.3 (0.8–1.8), control group: 1.2 (0.9–1.7)	Intervention group: 1.4 (1.0–2.0), control group: 1.4 (1.1–1.9)	Intervention group: 1.1 (0.9–1.5), control group: 1.0 (0.7–1.9)	Intervention group: 1.1 (0.9–1.5), control group: 1.0 (0.7–1.9)	Intervention group: 1.3 (1.0–1.6), control group: 1.0 (0.7–1.9)
24-h UACR (g/g)	Intervention group: 1.0 (0.7–1.5), control group: 1.1 (0.7–1.5)	-	Intervention group: 1.1 (0.8–1.5), control group: 1.1 (0.8–1.5)	Intervention group: 0.8 (0.6–1.3), control group: 0.7 (0.5–1.5)	Intervention group: 1.0 (0.8–1.2), control group: 0.7 (0.5–1.5)	Intervention group: 1.1 (0.8–1.4), control group: 0.7 (0.5–1.5)
eGFR (mL/min/1.73 m^2^), mean ± SD	Intervention group: 56.9 ± 24.4, control group: 57.1 ± 23.6	Intervention group: 56.8 ± 24.3, control group: 57.1 ± 23.6	Intervention group: 58.58 ± 23.75, control group: 59.49 ± 24.42	Intervention group: 74 ± 27, control group: 71 ± 24	Intervention group: 72 ± 24, control group: 71 ± 24	Intervention group: 70 ± 23, control group: 71 ± 24

BMI, body mass index; SBP, systolic blood pressure; DBP, diastolic blood pressure; UACR, urine albumin–creatinine ratio.

### Bias risk assessment

All four of the studies were double-blind RCTs [13, 16, 17, 20]. The random sequences of all studies were generated by computer software, and the assignment of the trial group was not known to all patients and practitioners except the data monitoring committee, so there was no significant selection bias, performance bias and detection bias. In addition, although individual patients withdrew from the study, the reasons for withdrawal were clear and the number of patients who dropped out did not affect the outcome analysis, so there was no significant attrition bias. Simultaneously, there was no evidence of selective reporting in any of the included studies. Therefore, the quality of all included studies is very high, which effectively ensures the reliability of this meta-analysis (Supplementary data, Table S1).

### Effects of treatment on UPCR

**Figure 2:**
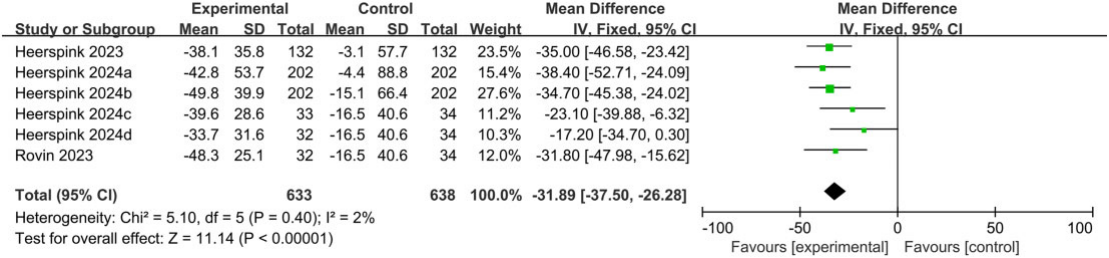
Comparison of the change from baseline in UPCR between the EARAs groups and the control group.

As shown in Fig. 2, the percentage reduction of UPCR from baseline in the EARAs groups was significantly higher than that in the control group (MD –31.89, 95% CI –37.50 to –26.28, *P* < .00001). Consistently, the proportion of patients getting UPCR decline ≥30%, ≥40% and ≥50% from baseline in the EARAs groups was higher than those in the control group (OR 2.58, 95% CI 0.98 to 6.76, *P* = .05, Supplementary data, Fig. S1A; OR 2.51, 95% CI 0.88 to 7.16, *P* = .08, Supplementary data, Fig. S1B; OR 6.45, 95% CI 3.27 to 12.70, *P* < .00001, Supplementary data, Fig. S1C; respectively), even though there was no significant difference in the proportion of patients getting UPCR reduction ≥30% and ≥40%.

### Effects of treatment on proteinuria remission

Supplementary data, Fig. S2 presented the proportion of patients getting complete and partial proteinuria remission in the EARAs groups and control group after treatment. Compared with control group, EARAs group achieved more complete proteinuria remission (OR 3.27, 95% CI 2.19 to 4.88, *P* < .00001, Supplementary data, Fig. S2A) and partial proteinuria remission (OR 3.28, 95% CI 2.43 to 4.42, *P* < .00001, Supplementary data, Fig. S2B).

### Effects of treatment on eGFR

**Figure 3:**
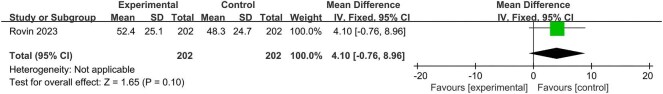
Comparison of the levels of eGFR after treatment between the EARAs groups and the control group.

As shown in Fig. 3, after treatment, the level of eGFR in the EARAs groups was higher than that in the control group, even though there was no statistical difference (MD 4.10, 95% CI –0.76 to 8.96, *P* = .10). Consistent with this, although another study including three RCTs were excluded from the meta-analysis because the relevant data could not be fully extracted [17], it was still observed that the reduction of eGFR from baseline in the EARAs groups was lower than that in the control group (the MDs versus control group in change from baseline were 1.1, 2.2 and 0.5 mL/min/1.73 m^2^ for the three EARAs groups, respectively).

### Effects of treatment on composite kidney failure endpoint

Compared with control treatment, EARAs resulted in fewer patients entering the composite kidney failure endpoint, although statistical significance was not reached (OR 0.61, 95% CI 0.36 to 1.04, *P* = .07, Supplementary data, Fig. S3).

### Effects of treatment on BP

**Figure 4:**
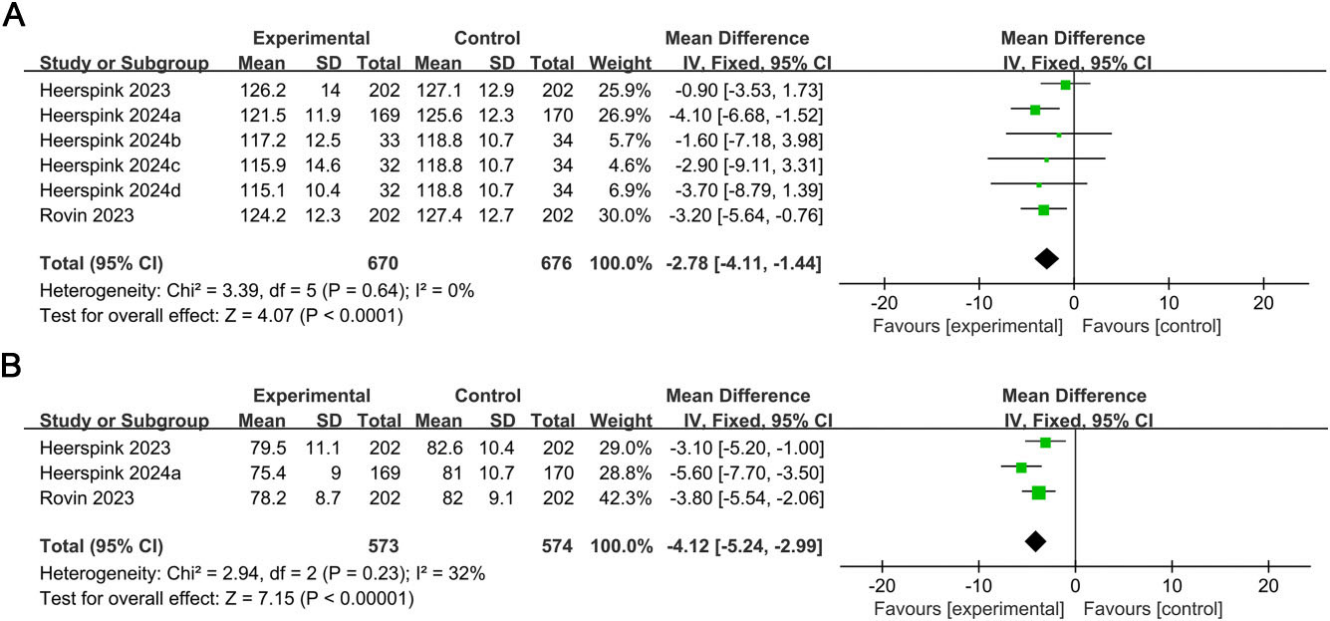
(**A**) Comparison of the levels of systolic BP after treatment between the EARAs groups and the control group; (**B**) comparison of the levels of diastolic BP after treatment between the EARAs groups and the control group.

As presented in Fig. 4, after treatment, both systolic and diastolic BP in the EARAs groups were lower than in the control group (MD –2.78, 95% CI –4.11 to –1.44, *P* < .0001, Fig. 4A; MD –4.12, 95% CI –5.24 to –2.99, *P* < .00001, Fig. 4B; respectively).

### Drug safety

#### Influences on hemoglobin, serum potassium, body weight and BNP

The influences of EARAs on hemoglobin, serum potassium, body weight and BNP were presented in Supplementary data, Fig. S4. Compared with control group, there was no significant change in hemoglobin and serum potassium after EARAs treatment (MD –2.08, 95% CI –8.54 to 4.39, *P* = .53, Supplementary data, Fig. S4A; MD 0.00, 95% CI –0.06 to 0.06, *P* = 1.00, Supplementary data, Fig. S4B; respectively). Although the statistical difference did not reach, the body weight gain from baseline in the EARAs groups was lower than that in the control group (MD –0.26, 95% CI –0.58 to 0.06, *P* = .11, Supplementary data, Fig. S4C). Moreover, the increase of BNP from baseline in the EARAs groups was higher than that in the control group, even if there was no statistical difference (MD 4.60, 95% CI 0.02 to 9.18, *P* = .05, Supplementary data, Fig. S4D). In line with this, although another study including three RCTs was excluded from this meta-analysis due to the fact that the standard deviation data could not be effectively extracted, we could still observe that EARAs may increase the level of BNP (the median change from baseline for the three EARAs groups and the control group was 0, 0.6, 17.1 and 0 pg/mL, respectively) [17].

#### TEAEs

As shown in Table 2, the overall incidence of TEAEs in the EARAs groups was similar to that in the control group (OR 1.25, 95% CI 0.92 to 1.70, *P* = .15), and no case of death was reported in the EARAs groups. However, anemia (OR 2.38, 95% CI 1.54 to 3.69, *P* = .0001), cough (OR 2.27, 95% CI 1.24 to 4.15, *P* = .008), dizziness (OR 2.37, 95% CI 1.51 to 3.71, *P* = .0002), hypotension (OR 2.39, 95% CI 1.56 to 3.67, *P* < .0001), fluid retention (OR 1.46, 95% CI 1.04 to 2.05, *P* = .003) and acute kidney injury (OR 3.12, 95% CI 1.31 to 7.42, *P* = .001) were more common in the EARAs groups than in the control group.

**Table 2:** Summary of TEAEs.

TEAEs	Studies	EARA groups (*n*/*N*)	Control group (*n*/*N*)	OR (95% CI)	*P*-value	Heterogeneity
Any TEAEs	6	584/669	572/676	1.25 (0.92, 1.70)	.15	*P* = .08, *I*^2^ = 49%
COVID-19	3	107/573	102/574	1.06 (0.79, 1.44)	.69	*P* = .76, *I*^2^ = 0%
Nasopharyngitis	6	51/669	40/676	1.31 (0.85, 2.01)	.22	*P* = .66, *I*^2^ = 0%
Anemia	6	69/669	32/676	2.38 (1.54, 3.69)	.0001	*P* = .57, *I*^2^ = 0%
Upper respiratory tract infection	6	64/669	49/676	1.37 (0.93, 2.02)	.11	*P* = .41, *I*^2^ = 1%
Headache	3	58/573	61/574	0.95 (0.65, 1.39)	.78	*P* = .90, *I*^2^ = 0%
Muscle spasms	3	36/573	34/574	1.06 (0.66, 1.73)	.80	*P* = .24, *I*^2^ = 30%
Cough	3	35/573	16/574	2.27 (1.24, 4.15)	.008	*P* = 1.00, *I*^2^ = 0%
Diarrhea	3	30/573	41/574	0.72 (0.44, 1.17)	.18	*P* = .34, *I*^2^ = 7%
Dizziness	3	66/573	30/574	2.37 (1.51, 3.72)	.0002	*P* = .67, *I*^2^ = 0%
Hypotension	3	73/573	33/574	2.39 (1.56, 3.67)	<.0001	*P* = .31, *I*^2^ = 16%
Nausea	2	19/371	12/372	1.62 (0.77, 3.39)	.20	*P* = .55, *I*^2^ = 0%
Back pain	3	28/573	45/574	0.60 (0.37, 0.98)	.04	*P* = .82, *I*^2^ = 0%
Hypertension	3	41/573	60/574	0.62 (0.31, 1.22)	.17	*P* = .13, *I*^2^ = 52%
Peripheral edema	3	94/573	68/574	1.46 (1.04, 2.05)	.03	*P* = .91, *I*^2^ = 0%
Hyperkalaemia	2	59/404	47/404	1.30 (0.86, 1.96)	.21	*P* = .92, *I*^2^ = 0%
Fatigue	2	32/404	19/404	1.74 (0.97, 3.13)	.06	*P* = .74, *I*^2^ = 0%
Blood creatine phosphokinase increased	2	25/404	18/404	1.42 (0.76, 2.64)	.27	*P* = .76, *I*^2^ = 0%
Arthralgia	2	24/404	23/404	1.05 (0.58, 1.89)	.88	*P* = .90, *I*^2^ = 0%
Proteinuria	2	22/404	14/404	1.61 (0.81, 3.19)	.18	*P* = .56, *I*^2^ = 0%
Acute kidney injury	2	21/404	7/404	3.12 (1.31, 7.42)	.01	*P* = .51, *I*^2^ = 0%
Pruritus	2	22/404	13/404	1.73 (0.86, 3.49)	.12	*P* = .50, *I*^2^ = 0%
Blood creatinine increased	2	27/404	35/404	0.75 (0.45, 1.27)	.29	*P* = 0.31, *I*^2^ = 4%
Urinary tract infection	4	13/298	24/304	0.53 (0.27, 1.07)	0.08	*P* = .88, *I*^2^ = 0%
Aminotransferase elevations	5	31/500	37/506	0.85 (0.51, 1.40)	.52	*P* = .38, *I*^2^ = 4%
Hyperuricemia	4	30/298	27/304	1.15 (0.67, 1.98)	.62	*P* = .34, *I*^2^ = 10%
Hyperlipidemia	3	11/96	18/102	0.60 (0.27, 1.35)	.22	*P* = .50, *I*^2^ = 0%
Weight decreased	3	5/96	9/102	0.57 (0.18, 1.76)	.32	*P* = .88, *I*^2^ = 0%
Lipase increased	2	22/404	14/404	1.61 (0.81, 3.19)	.18	*P* = .56, *I*^2^ = 0%
Others	6	63/669	68/676	0.92 (0.64, 1.33)	.66	*P* = .69, *I*^2^ = 0%

*n*, the number of patients who experienced TEAEs; *N*, the total number of patients included.

#### Publication bias assessment

Egger's test and Begg-Mazumdar test were used to assess the publication bias, and the percentage reduction of UPCR from baseline was used as the effect index. As presented in Supplementary data, Fig. S5, no publication bias was observed by Egger's test (*P* = .500) and Begg-Mazumdar test (*P* = .133).

## DISCUSSION

In this meta-analysis, we systematically evaluated the efficacy and safety of EARAs in primary IgAN patients for the first time, based on four high-quality studies including 1346 patients.

As we know, proteinuria is the most widely recognized and studied risk factor for progression to ESRD in IgAN, and proteinuria reduction has been considered to be a surrogate endpoint for effective treatment of IgAN [21–23]. Therefore, the effect of EARAs on UPCR was explored in detail in our meta-analysis. Compared with control treatment, IgAN patients in the EARAs groups had a significant reduction in the UPCR by 31.89 percentage points. This benefit is very valuable for IgAN patients, especially for those who are at high risk of disease progression. Similarly, Nagasawa *et al*. found that saprsentan was superior to losartan in reducing albuminuria in the gddY mouse model of IgAN [24]. King *et al*. found that atrasentan could significantly reduce albuminuria in the gddY mouse model of spontaneous IgAN [25]. Moreover, in our meta-analysis, the proportion of patients achieving UPCR decline ≥30%, ≥40% or ≥50% from baseline, and the proportions of patients achieving complete or partial proteinuria remission in the EARAs groups were all higher than in the control group. All these results suggest that EARAs can effectively reduce the level of urinary protein excretion in IgAN patients.

Like proteinuria, GFR decline is another independent risk factor for progression to ESRD in IgAN, and delaying the decline of eGFR is also an acceptable surrogate endpoint for effective treatment of IgAN patients [4, 23]. Therefore, the effect of EARAs on eGFR was also evaluated in detail. As expected, the reduction of eGFR from baseline in the EARAs groups was lower than that in the control group. This result is supported by the study of Heerspink *et al*. [26], who found that zibotentan in combination with dapagliflozin was more effective than dapagliflozin alone in delaying the decline of eGFR in patients with chronic kidney disease (CKD), including IgAN. Similarly, Nakamura *et al*. found that FR 139317 (one kind of EARA) could significantly delay the decline of eGFR in ddY mice with IgAN [14]. Moreover, compared with control group, we found that fewer IgAN patients in the EARAs groups reached the composite kidney failure endpoint in our meta-analysis, which further indicates that EARAs can effectively delay the decline of eGFR in IgAN patients.

As another recognized independent risk factor for progression to ESRD in IgAN, the changes in BP before and after treatment were also carefully explored. In our meta-analysis, we found that EARAs could significantly lower both systolic and diastolic BP in IgAN patients. Similarly, Nagasawa *et al*. found that, compared with losartan, sparsentan could significantly reduce the level of systolic BP in gddY mouse model of IgAN [24]. Heerspink *et al*. also observed that zibotentan combined with dapagliflozin was more effective than dapagliflozin alone in reducing both systolic and diastolic BP in CKD patients, including IgAN patients [26]. Furthermore, EARAs have been widely recommended for the treatment of pulmonary arterial hypertension [27–29]. All these results indicate that EARAs can effectively reduce the level of BP in IgAN patients.

In addition to the therapeutic effects, the safety of EARAs in IgAN patients was also evaluated in detail. As mentioned above, the overall incidence of TEAEs in the EARAs groups was similar to that in the control group, and no fatal case was reported in the EARAs groups, suggesting that the safety of EARAs is reliable. However, compared with control group, fluid retention was more common in the EARAs groups in our meta-analysis. Consistently, Heerspink *et al*. found that zibotentan in combination with dapagliflozin was more likely to cause fluid retention than dapagliflozin alone [26]. Wei *et al*. conducted a meta-analysis of 24 RCTs involving 4894 patients and found that patients receiving EARAs treatment were more likely to develop peripheral edema [30]. Interestingly, Yu *et al*. found that fluid retention caused by EARAs might be an adaptive response of the body, aiming to maintain cardiac filling and cardiac output [31]. Therefore, it is suggested that fluid retention may be a common adverse reaction of EARAs in IgAN patients.

In addition, we found an increase in BNP levels after EARAs treatment in our meta-analysis. Since EARAs can cause fluid retention, it may increase the risk of developing heart failure. In fact, EARAs might be indeed associated with an increased risk of heart failure [15, 32]. In particular, Smeijer *et al*. have found that increased BNP after atrasentan treatment was associated with incident heart failure [33]. Therefore, IgAN patients with an underlying history of heart failure may need to use these drugs with caution.

Moreover, body weight loss was observed after EARAs treatment in our meta-analysis, even though only slight. Considering that EARAs might be associated with fluid retention, and body weight gain is a marker of fluid retention, theoretically EARAs should be related to body weight gain. In fact, Heerspink *et al*. have found that zibotentan might be associated with body weight gain in CKD patients, including IgAN patients [26]. Similarly, Schievink *et al*. also found that atrasentan caused body weight gain in patients with diabetic kidney disease [34]. The reason for this contradictory result is not clear—we hypothesize that differences in disease types may be a potential reason, as the population that Schievink *et al*. studied was predominantly patients with diabetic kidney disease, and more research is needed to further explore the effect of EARAs on body weight in IgAN patients.

As previously mentioned, EARAs could help slow down the decline of eGFR in IgAN patients, but we still observed that acute kidney injury was more common in patients receiving EARAs treatment in our meta-analysis. Similarly, Dhaun *et al*. also found that sitaxsentan could reduce both glomerular rate and filtration fraction, even though there was no clinically significant adverse event [35]. Studies found that, similar to renin–angiotensin system inhibitors, EARAs could block the binding of endothelin-1 to endothelin A receptor, subsequently preventing the constriction of the efferent arteriole induced by endothelin-1, and leading to a decrease in glomerular perfusion pressure. This would result in a short-term decrease in eGFR [35–37]. Therefore, we speculate that the occurrence of acute kidney injury in patients after EARAs treatment may be related to this mechanism. However, combining the overall impact of EARAs on eGFR observed in our meta-analysis and literature reports, we believe that eGFR may still benefit in the long term, even though patients may experience a decrease in eGFR after EARAs treatment in the short term.

Additionally, although EARAs had no significant effect on hemoglobin in our meta-analysis, anemia was still more common in the EARAs groups than control group. Similarly, Wei *et al*. found that patients receiving EARAs treatment were more likely to suffer from anemia [30]. We speculate that the development of anemia secondary to hemodilution, as a result of fluid retention induced by EARAs, may be a potential mechanism, which is consistent with the speculation of Wei *et al*. [30] Moreover, compared with the control group, hypotension was more common after EARAs treatment in our meta-analysis. Given that EARAs could lower both systolic and diastolic BP in IgAN and non-IgAN patients [26, 38–40], it is necessary to closely monitor patients’ BP during medication.

Notably, although both included studies reported that patients in the EARAs groups were more likely to experience adverse reaction of hyperkalemia than those in the control group [16, 20], we found that there was no significant difference in the incidence of hyperkalemia between the EARAs groups and control group in our meta-analysis. Consistent with this, other published study has also not found that EARAs could cause hyperkalemia in patients with CKD, including IgAN [26]. Meanwhile, no significant effect of EARAs on levels of blood potassium was observed in our meta-analysis. Therefore, it is suggested that EARAs may have no significant influence on blood potassium in IgAN patients. Moreover, other notable adverse reactions induced by EARAs included cough and dizziness, which is consistent with the findings of other studies [41, 42].

### Limitations

Although all the included studies in our meta-analysis were high-quality, double-blind RCTs, which could provide strong supports for the results, there were still some limitations. First, the number of included studies was small, which may affect the results due to insufficient data collection. Second, the heterogeneity of treatment may influence the results, including the difference in drug type, dosage and duration of follow-up. Third, the differences in population characteristics may affect the results, including difference in gender, age and duration of disease. Four, the mean and/or SD values of eGFR and BNP from one study could not be effectively extracted [17], which may affect the final analysis of these two outcomes.

## CONCLUSION

In conclusion, compared with control treatment, EARAs could significantly reduce UPCR, lower both systolic and diastolic BP, and slow down the decline of eGFR in IgAN patients. The overall safety of EARAs was reliable. Notably, EARAs did not have a significant effect on hemoglobin and blood potassium, but may be associated with body weight loss and BNP increase. Moreover, it should be noted that EARAs may cause anemia, cough, dizziness, hypotension, fluid retention and acute kidney injury. More high-quality, large-sample RCTs are required to further confirm the long-term efficacy and safety of EARAs in IgAN patients.

## Supplementary Material

sfaf066_Supplemental_File

## Data Availability

All data that support the findings appear in the article.
