# Supplementary material for: Efficacy and safety of endothelin A receptor antagonists in IgA nephropathy: a systematic review and meta-analysis
Source: Clin Kidney J. 2025 Feb 27;18(3):sfaf066. doi: 10.1093/ckj/sfaf066 (PMC11932335; doi:10.1093/ckj/sfaf066)
Supplement: sfaf066_Supplemental_File [file sfaf066_supplemental_file.pdf]

Supplementary Materials

Supplementary Figure S1 A. Comparison of the proportion of patients with ≥30% reduction from baseline in UPCR between EARAs group and control group; B. Comparison of the proportion of patients with ≥40% reduction from baseline in UPCR between EARAs group and control group; C. Comparison of the proportion of patients with ≥50% reduction from baseline in UPCR between EARAs group and control group

Supplementary Figure S2 A. Comparison of the proportion of patients getting complete proteinuria remission between EARAs group and control group; B. Comparison of the proportion of patients getting partial proteinuria remission between EARAs group and control group

Supplementary Figure S3 Comparison of the proportion of patients reaching composite kidney failure endpoint between EARAs group and control group

Supplementary Figure S4 A. Comparison of the change from baseline in hemoglobin between EARAs group and control group; B. Comparison of the levels of serum potassium after treatment between EARAs group and control group; C. Comparison of the change from baseline in body weight between EARAs group and control group; D. Comparison of the change from baseline in BNP between EARAs group and control group

Supplementary Figure S5 A. Egger's publication bias plot; B. Begg's funnel plot

Supplementary Table S1 Bias risk assessment of included studies

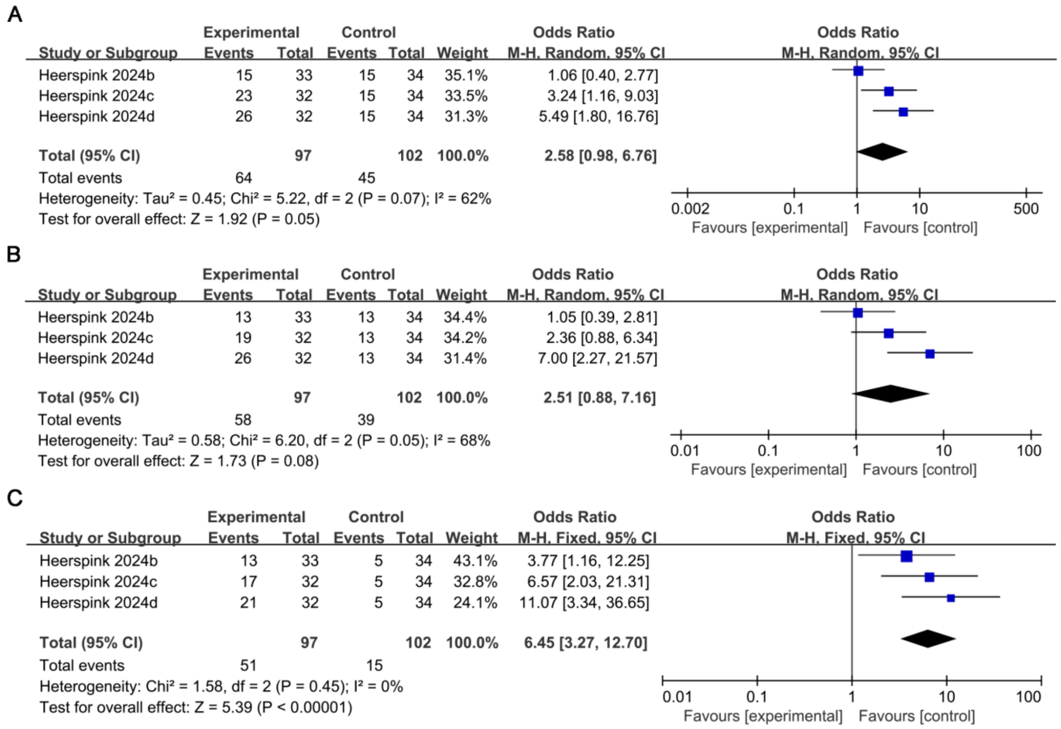

Supplementary Figure S1 A. Comparison of the proportion of patients with ≥30% reduction from baseline in UPCR between EARAs group and control group; B. Comparison of the proportion of patients with ≥40% reduction from baseline in UPCR between EARAs group and control group; C. Comparison of the proportion of patients with ≥50% reduction from baseline in UPCR between EARAs group and control group

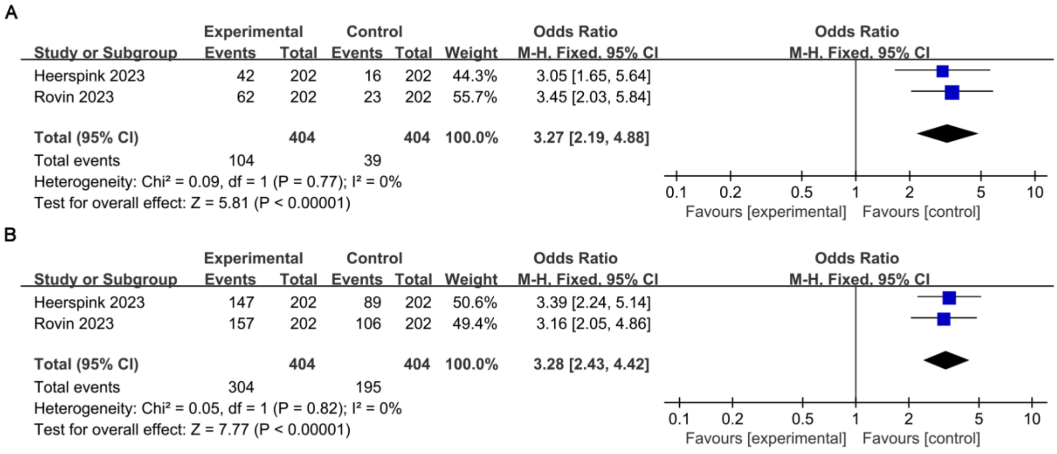

Supplementary Figure S2 A. Comparison of the proportion of patients getting complete proteinuria remission between EARAs group and control group; B. Comparison of the proportion of patients getting partial proteinuria remission between EARAs group and control group

Study or Subgroup	Experimental Events	Experimental Total	Control Events	Control Total	Weight	Odds Ratio M-H, Fixed, 95% CI
Heerspink 2023	7	202	13	202	34.6%	0.52 [0.20, 1.34]
Rovin 2023	18	202	26	202	65.4%	0.66 [0.35, 1.25]
**Total (95% CI)**		**404**		**404**	**100.0%**	**0.61 [0.36, 1.04]**
Total events	25		39			

Heterogeneity: Chi² = 0.17, df = 1 (P = 0.68); I² = 0%

Test for overall effect: Z = 1.82 (P = 0.07)

Odds Ratio
M-H, Fixed, 95% CI

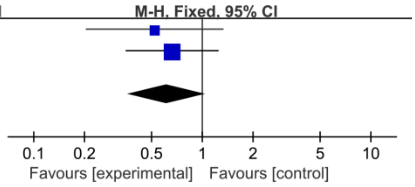

Supplementary Figure S3 Comparison of the proportion of patients reaching composite kidney failure endpoint between EARAs group and control group

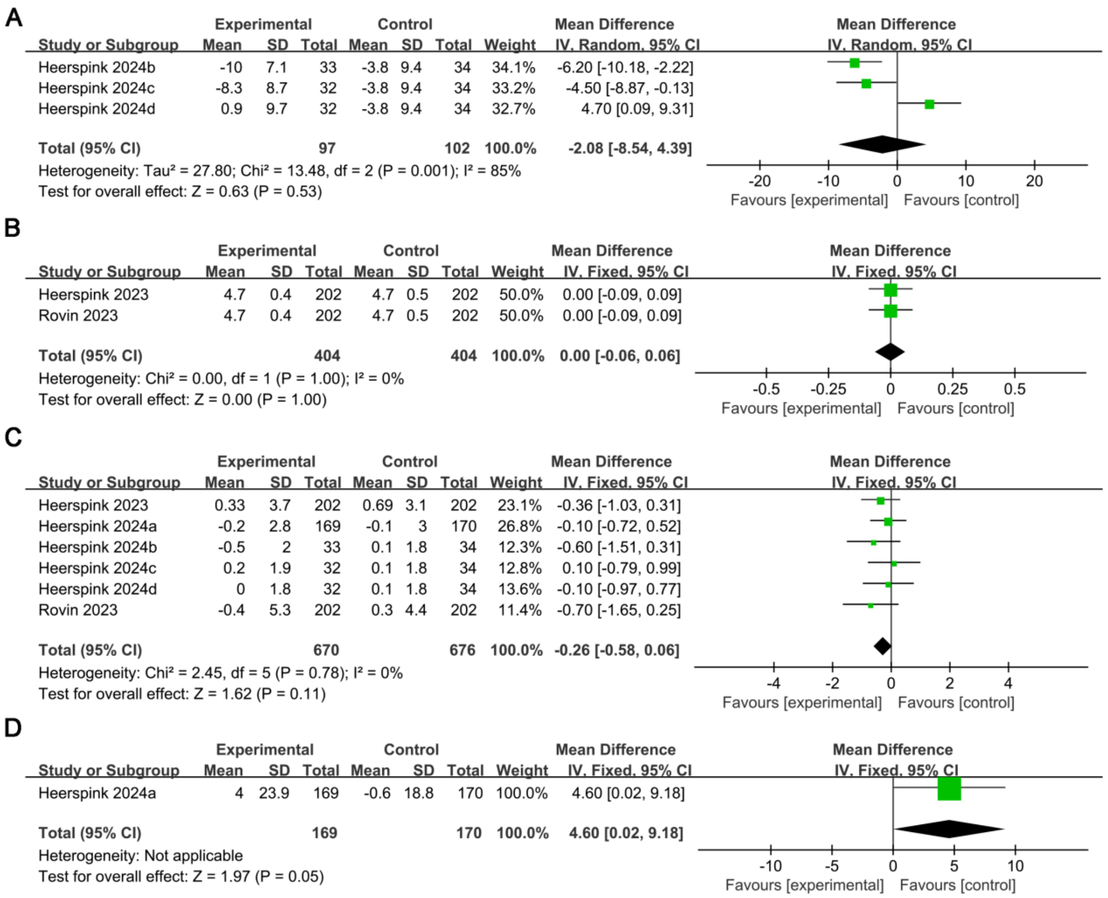

Supplementary Figure S4 A. Comparison of the change from baseline in hemoglobin between EARAs group and control group; B. Comparison of the levels of serum potassium after treatment between EARAs group and control group; C. Comparison of the change from baseline in body weight between EARAs group and control group; D. Comparison of the change from baseline in BNP between EARAs group and control group

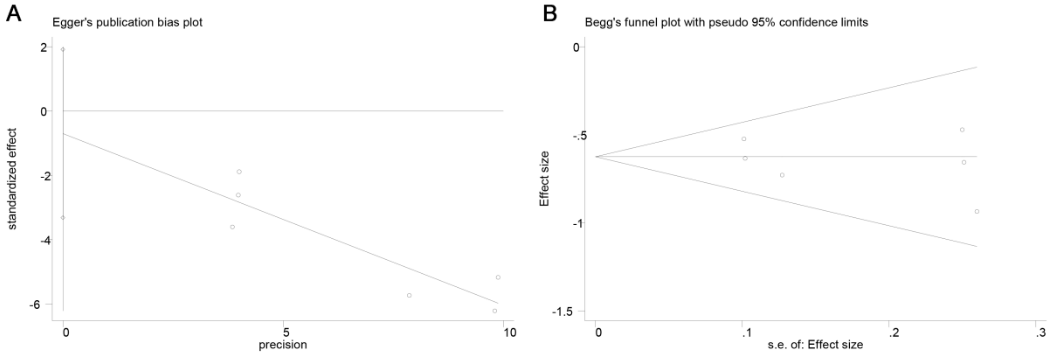

Supplementary Figure S5 A. Egger's publication bias plot; B. Begg's funnel plot

Supplementary Table S1 Bias risk assessment of included studies

Study	Random sequence generation (selection bias)	Allocation concealment (selection bias)	Blinding of participants and personnel (performance bias)	Blinding of outcome assessment (detection bias)	Incomplete outcome data (attrition bias)	Selective reporting (reporting bias)
Heerspink 2023[1]	low	low	low	low	low	low
Rovin 2023[2]	low	low	low	low	low	low
Heerspink 2024a[3]	low	low	low	low	low	low
Heerspink 2024b[4]	low	low	low	low	low	low
Heerspink 2024c[4]	low	low	low	low	low	low
Heerspink 2024d[4]	low	low	low	low	low	low

References

1. Heerspink HJL, Radhakrishnan J, Alpers CE et al. Sparsentan in patients with IgA nephropathy: a prespecified interim analysis from a randomised, double-blind, active-controlled clinical trial. Lancet 2023;**401**:1584-1594. https://doi.org/10.1016/S0140-6736(23)00569-X
2. Rovin BH, Barratt J, Heerspink HJL et al. Efficacy and safety of sparsentan versus irbesartan in patients with IgA nephropathy (PROTECT): 2-year results from a randomised, active-controlled, phase 3 trial. Lancet 2023;**402**:2077-2090. https://doi.org/10.1016/S0140-6736(23)02302-4
3. Heerspink HJL, Jardine M, Kohan D E et al. Atrasentan in patients with IgA nephropathy. N Engl J Med 2024:1-11. https://doi.org/10.1056/NEJMoa2409415
4. Heerspink HJL, Du X, Xu Y et al. The selective endothelin receptor antagonist SC0062 in IgA nephropathy: a randomized double-blind placebo-controlled clinical trial. J Am Soc Nephrol 2024:1-11. https://doi.org/10.1681/ASN.0000000538
